# Formation of *N*^ε^-Carboxymethyl-Lysine and *N*^ε^-Carboxyethyl-Lysine in Pacific Oyster (*Crassostrea gigas*) Induced by Thermal Processing Methods

**DOI:** 10.3389/fnut.2022.883789

**Published:** 2022-04-15

**Authors:** Pengcheng Zhou, Shiyuan Dong, Mingyong Zeng

**Affiliations:** College of Food Science and Engineering, Ocean University of China, Qingdao, China

**Keywords:** advanced glycation end products, *N*^ε^-carboxymethyl-lysine, *N*^ε^-carboxyethyl-lysine, thermal processing, pacific oyster, sous vide

## Abstract

Advanced glycation end products (AGEs) are important endogenous hazardous substances produced during the thermal processing of foods, which have attracted much attention due to the potential health risks. The current research first investigated the effect of different thermal processing methods (steaming, boiling, sous vide (SV), and sterilizing) on the formation of two typical markers of AGEs, including *N*^ε^-carboxymethyl-lysine (CML) and *N*^ε^-carboxyethyl-lysine (CEL), in Pacific oyster (*Crassostrea gigas*). The compositions, lipid oxidation, di-carbonyl compounds, and AGEs in 12 kinds of processed oysters were detected, and the Index values (total *Z*-score) were calculated. The SV treatment at 70°C caused higher processing yield and lower CEL level while sterilizing in oil at 121°C greatly resulted in the formation of CML. The Index value of SV-treated oysters was much lower than steamed, boiled, and sterilized ones. Correlation analysis showed that the CML and CEL levels were positively correlated with fat content, a^*^ and b^*^ value (*p* < 0.05), and negatively correlated with moisture content and L^*^ value (*p* < 0.05). Besides, thiobarbituric acid reactive substances had a negative correlation with CML (*r* = −0.63, *p* < 0.05) while no significant correlation with CEL (*p* > 0.05), suggesting that lipid oxidation had a greater effect on the formation of CML but less on the formation of CEL. In summary, SV treatment at 70°C within 15 min was a recommended thermal processing method to reduce the formation of AGEs in oysters.

## Introduction

Many physical and chemical changes affect the quality of food during thermal processing. Proper thermal processing could eliminate the harm of pathogenic microorganisms and improve food's flavor, texture, color, and nutritional value (1). The Maillard reaction is one of the critical reactions in the thermal processing of food, which produces tantalizing aroma and color. It generates a series of complex chemicals at the advanced stage named advanced glycation end products (AGEs) (2). High levels of AGEs in the daily diet led to the retention and accumulation in the human blood, which were reported to induce diabetes (3), kidney disease (4), cardiovascular diseases (5), and inflammation (6). In addition, long-term consumption of food with high di-carbonyl compounds could also elevate the level of di-carbonyl compounds in human plasma and lead to severe endothelial dysfunction (7). *N*^ε^-carboxymethyl-lysine (CML) and *N*^ε^-carboxyethyl-lysine (CEL) are typically two kinds of AGEs biomarkers in food (8). The Amadori rearrangement products generated in the early stage of the Maillard reaction are dehydrated and rearranged to produce highly reactive di-carbonyl compounds, such as glyoxal (GO) and methylglyoxal (MGO), which react with lysine residues to form CML and CEL (9). Besides, GO, and MGO could also be generated by the oxidation pathways of lipids and reducing sugars, eventually forming CML and CEL (10).

To the best of our knowledge, the formation of CML and CEL depends on food composition, processing time, and temperature. Some studies have determined the contents of CML and CEL in hundreds of food products by ultra-performance liquid chromatography-tandem mass spectrometry (UPLC–MS/MS) and established a reliable dietary AGE database (11, 12). The results showed that foods with high protein or fat content, such as milk, cheese, biscuits, and chocolates, had relatively high AGEs. In contrast, foods with high moisture content, such as apple, mushroom, and tomato soup, had relatively low AGEs. Recently, more and more studies have focused on the effects of different thermal processing on the formation of AGEs. For instance, Sun et al. (13) found that (121°C for 10 min) led to about 0.6- to 3.6-fold increase of CML and CEL in beef, pork, or chicken. Yu et al. (14) showed that the content of CML in pasteurized meat products was 1.5-fold higher than that in sterilized ones, but the content of CEL in sterilized meat products was 2.5-fold higher than that in pasteurized ones. Our previous study found that the CML levels in fried hairtail filets were higher than boiled and baked ones, regardless of the cooking time (15).

Oysters are one of the largest marine bivalves for global human consumption. The global production of oysters has reached >5,000,000 t, and China produces about 82% of the total output (16). Pacific oysters (*Crassostrea gigas*) are the main cultured oyster species in North China (Shandong and Liaoning provinces), with an annual production of >1,200,000 t (17). In China and other Asian countries, steaming and boiling are the most common home cooking methods of oysters, and commercial sterilization is used in the industrial production of canned oysters (18). In addition, sous vide (SV) treatment is an emerging method of low-temperature and long-time heating in a thermostable vacuum bag, which reduces moisture loss and oxidation of asparagus spears (19), beef (20), and fish burgers (21). However, there is little information about the profiles of CML and CEL in the thermally processed oyster.

The objective of this study was primarily to compare the generation of CML and CEL at four thermal processing methods (steaming, broiling, sous vide, and sterilizing) of Pacific oysters (*Crassostrea gigas*). Particularly, the compositions, lipid oxidation products, di-carbonyl compounds, and AGEs were investigated, and the correlation between these parameters was analyzed. Index value (total *Z*-score) of the processed oysters was also calculated to elucidate the effect of different methods on the formation of CML and CEL. The findings of this study could help optimize the thermal processing conditions of oysters to minimize CML and CEL formation.

## Materials and Methods

### Materials

Fresh Pacific oyster (*Crassostrea gigas*) was purchased in October from Qingdao (Shandong province, China) aquatic product market and transported to the laboratory immediately on ice. CML, CEL, *N*^ε^-carboxymethyl-lysine-d4 (CML-d4) and *N*^ε^-carboxyethyl-lysine-d4 (CEL-d4) were obtained from Toronto Research Chemicals Inc. (Toronto, Canada). GO, MGO, and o-Phenylenediamine (OPD) were bought from Sigma-Aldrich (St. Louis, MO, USA). Acetonitrile and methanol of HPLC grade were purchased from Merck (Darmstadt, Germany). All other chemical reagents used in this study were of analytical grade.

### Sample Preparation

Oysters were treated in various ways, reflecting the traditional and emerging thermal treatments of shellfish. The 12 treatments included the following: raw sample (Control); steaming at 98°C for 5 min (ST5), 10 min (ST10), and 15 min (ST15) (mimicking steaming of shelled oyster); boiling at 98°C for 5 min (BO5), 10 min (BO10), and 15 min (BO15) (mimicking boiling of oyster meat); sous vide processing at 70°C for a time when the internal temperature reached 70°C and stayed at this temperature for 5 min (SV5), 10 min (SV10), and 15 min (SV15) (used as a new method for cooking seafood at French restaurants) (22); sterilizing in water (SW30) or oil (SO30) at 121°C for 30 min (mimicking sterilization of canned oyster). Photos of raw and processed oyster meats are shown in Supplementary Table 1. Oysters processed at 70 and 121°C were vacuum-packed in retort pouches (20 cm × 15 cm, PA/PE, Leadpacks Environmental Protection Packing Co., Ltd, Fujian, China). A digital probe thermometer (NAPUI thermocouple, Mod.TR230X-8, Guangdong, China) was used to measure the internal temperature of oysters during heating. The above preparation of samples was triplicate. After thermal processing, the steamed and sous vide treated oysters were shucked, packed, and plunged into iced water to cool below 20°C within 5 min. Oyster meats were mixed with deionized water at a ratio of 1:3 (w/v) and then freeze-dried. Freeze-dried samples were kept at −25°C within 3 months for further analyses.

### Processing Yield, Moisture, Protein, Lipids, and Lysine Measurement

The processing yield was calculated following the USDA (2012) formula:


(1) $$\begin{array}{r} Yield\;\left(\%\right)=\frac{Processed\;sample\;weight}{Raw\;sample\;weight}\times 100\; \end{array}$$

The content of moisture, protein, and lipids in oyster meats was analyzed by the AOAC official methods (23). The content of lysine was determined using an amino acid analyzer (L-8500A, Hitachi Co., Tokyo, Japan) according to Cao et al. (24).

### Color Measurement

According to the method slightly modified by Tavares et al. (15), the surface color of raw and processed oysters was measured by the chroma meter (3nh, NR60CP, Guangdong, China) with a viewing angle of 10° and an illuminant D-65. Before testing, the equipment was calibrated against a standard whiteboard. Measurement for each sample after calibration, where L^*^ refers to the lightness component, a^*^ red (+a) to green (–a) component, and b^*^ yellow (+b) to blue (–b) component. ΔE (the total color difference) was determined by the following equation. The final result was the mean value of reading three times on the visceral mass of each sample.


(2) $$\begin{array}{r} \Delta E={\left[{\left(L_{s}-L_{0}\right)}^{2}+{\left(a_{s}-a_{0}\right)}^{2}+{\left(b_{s}-b_{0}\right)}^{2}\right]}^{\frac{1}{2}} \end{array}$$

Where “s” defines values of processed samples and “0” denotes values of raw samples.

### Lipid Oxidation Products Measurement

To evaluate the level of lipid oxidation in processed oysters, thiobarbituric acid-reactive substances (TBARS) were determined using UV–VIS spectrophotometer (UV-2550, Shimadzu, Japan). TBARS analysis was performed according to Wang et al. (25), and the absorbance of the resulting solution was measured at 532 nm against a blank containing only extraction and TBA solutions. The results of TBARS content were expressed as malondialdehyde (MAD) (mg MAD/kg dry sample).

### Analysis of di-Carbonyl Compounds

Glyoxal and MGO were determined as described by Kocadagli et al. (26, 27) with some modifications. The analyses were performed using an Agilent Series 1200 binary pump LC system (Agilent Technologies, Santa Clara, CA, USA), coupled to an Agilent triple quadrupole mass spectrometer (6410B) equipped with electrospray ionization. A Waters Symmetry C18 column (2.1 × 100 mm, 3.5 μm) was used.

### Analysis of CML and CEL

#### Sample Preparation

A modified acid hydrolysis method was employed to prepare CML and CEL analysis (28, 29). First, 0.02 g of raw or processed freeze-dried samples were incubated with 0.4 mL borate buffer (0.2 M, pH 9.2) and 0.08 ml sodium borohydride (2 M in 0.1 M NaOH) at 4°C for 8 h and then hydrolyzed with 0.8 ml 6 M HCl at 110°C for 24 h. Next, the protein hydrolysate was dried in a vacuum oven (DZF-6050; Shanghai Jinghong Laboratory instrument Co., Ltd, Shanghai, China) at 60°C and diluted with water to 4 mL, from which 1 ml were withdrawn and spiked with 20 μL CML-d4, CEL-d4 (5 μg/mL, internal standard). Following this, the sample solution was purified by a pre-activated MCX column (60 mg/3 ml; Shanghai ANPEL Scientific instrument Co., Ltd, Shanghai, China) and eluted with 3 ml methanol containing 5% ammonia water. Finally, the eluent was dried in nitrogen with a nitrogen evaporator (DC12H; Shanghai ANPEL Scientific Instrument Co., Ltd, Shanghai, China), reconstituted with 2 mL deionized water, and filtered through a 0.22 μm filter before LC-MS/MS analysis.

#### LC-MS/MS Analysis

The analysis of CML and CEL in the sample extracts was performed with a Waters 2695 HPLC system (Waters Inc., Milford, USA) and a Waters Quattro Micro triple-quadrupole tandem mass spectrometer (MS/MS) operated in positive electrospray ionization (ESI) mode. A Waters ACQUITY BEH Amide column (2.1 × 100 mm, 1.7 μm) was used. The sample injection volume was 3 μL, and the column temperature was set at 35°C. The binary mobile phase used was (A) water containing 10 mM ammonium formate and 0.1% formic acid, and (B) acetonitrile. The instrument settings for the mass spectrometer were the same as those described in the study of Sun et al. (28).

### Calculation of *Z*-Score and Index

According to the method of Guseman et al. (30), *Z*-score and Index value (total *Z*-score) were measured with a slight modification. In brief, the average concentration of the three repeats was used to calculate each sample's *Z*-score following Equation A and an Index value was created by following Equation B.


(3) $$\begin{array}{rcl} A:Z & = & X-\frac{x}{s} \end{array}$$

(4) $$\begin{array}{rcl} B:Index & = & Z_{GO}+Z_{MGO}+Z_{CML}+Z_{CEL} \end{array}$$

Where A: X = the average level of each sample, x = the mean of the 12 processed samples, and s = the standard deviation of the 12 processed samples. B: Z_GO_ = the sample's *Z*-score of GO, Z_MGO_ = the sample's *Z*-score of MGO, Z_CML_ = the sample's Z-score of CML, Z_CEL_ = the sample's *Z*-score of CEL.

### Statistical Analysis

The experiments were performed in triplicate, and results were expressed in means ± *SD*. The statistical analysis was performed using SPSS 22 software (SPSS Inc., Chicago, IL, USA). Significant differences in the means of treatments were identified at a level of *p* < 0.05 by one-way ANOVA and the Duncan test. Pearson's correlation test was analyzed by OriginPro 2021b software (OriginLab Inc., Northampton, MA, USA). *Z*-score analysis was conducted using Microsoft Excel 2016 software (Microsoft corp., Redmond, WA, USA).

## Results and Discussion

### Processing Yield and Composition

The internal temperature of all processed oysters was higher than the safe minimum internal temperature of 62.8°C (31), and the microbial safety could be guaranteed. The processing yield, moisture, protein, and fat contents of raw and processed oyster meats are shown in Table 1. Compared with raw oysters, the processing yield and moisture level decreased. Sterilizing in water (SW30) or oil (SO30) resulted in a processing yield of 52.35 and 48.3%, respectively. Conversely, the processing yield of SV-treated samples was above 86%, which was related to the processing temperature maintained at 70°C. The fat content in all processed oysters increased compared to the raw ones, and a significant difference was observed, especially in SO30 (*p* < 0.05). The fact was probably related that the moisture lost during sterilizing was replaced by oil uptake through capillary pores formed by water evaporation, which increased fat content (32). Besides, protein content is an important indicator to evaluate the quality of nutrition and safety in thermally processed oysters (33). Compared to the raw oysters, protein content in processed ones increased due to internal water losses (15). Interestingly, the protein content significantly decreased from 57.22% of raw oysters to 52.61% of SO30 (*p* < 0.05). According to Ismail et al. (34), the thermal processing method and duration greatly influenced the content of moisture, fat, and protein. Therefore, we suspect that the decrease of protein content may be due to the protein degradation during sterilizing in oil at 121°C for 30 min.

**Table 1:** Processing yield (%) and composition of raw and processed oysters (per 100 g of dry matter).

**Treatments**	**Time (min)**	**Processing yield (%)**	**Moisture (%)**	**Fat (% dry sample)**	**Protein (% dry sample)**
Raw	—	100.00	80.55 ± 0.07^a^	8.68 ± 0.34^g^	57.22 ± 1.01^e^
Steamed	5	75.60 ± 0.75	76.54 ± 0.63^cd^	9.81 ± 0.97^def^	60.78 ± 0.36^c^
	10	70.70 ± 0.50	75.25 ± 0.78^de^	10.30 ± 0.20^de^	64.25 ± 0.19^a^
	15	67.41 ± 0.35	72.46 ± 1.51^f^	10.85 ± 0.43^cd^	65.02 ± 0.66^a^
Boiled	5	73.62 ± 1.51	77.08 ± 0.31^c^	10.70 ± 0.00^cd^	60.78 ± 0.47^c^
	10	63.11 ± 1.59	76.25 ± 0.35^cd^	11.60 ± 0.35^c^	61.80 ± 0.71^b^
	15	58.69 ± 1.57	74.05 ± 0.64^e^	10.37 ± 0.56^de^	61.87 ± 0.73^b^
Sous vide^1^	5	92.10 ± 1.27	79.50 ± 0.57^ab^	9.91 ± 0.10^def^	58.56 ± 0.05^d^
	10	88.65 ± 0.92	79.05 ± 0.92^b^	8.98 ± 0.09^fg^	57.22 ± 0.02^e^
	15	86.00 ± 0.99	78.83 ± 0.46^b^	9.67 ± 0.48^efg^	58.12 ± 0.48^d^
Sterilized (in water)	30	52.35 ± 1.20	72.26 ± 0.78^f^	12.65 ± 0.31^b^	60.47 ± 0.06^c^
Sterilized (in oil)	30	48.30 ± 0.57	66.01 ± 1.84^g^	21.63 ± 1.45^a^	52.61 ± 0.09^f^

*Value are means ± SD (n = 3). Different lowercase letters in the same column indicate the significant difference (p < 0.05)*.

1 *Time indicates the internal temperature of the oyster reached 70°C and stayed at this temperature for 5, 10, and 15 min*.

### Color Values

The color values of oyster meat under different thermal processing conditions are illustrated in Table 2. The steamed, boiled, and SV-treated oysters indicated higher L^*^, a^*^, or b^*^value than the raw ones regardless of processing time. The a^*^ or b^*^ value of sterilized oysters was higher than the raw ones, while L^*^ value of raw oysters was lower than the sterilized ones. The a^*^ or b^*^ value in steamed and SV-treated oysters increased with processing time, while the opposite trend was observed in the boiled ones. The ΔE of SV-treated oysters from 6.16 to 8.52 was the lowest value in all four methods. Conversely, the ΔE value of SO30 (26.96) was much higher than the other processed samples. The reason behind this phenomenon was most likely due to oil infiltration and some colored substances formed on the surface of oyster meats through the Maillard reaction pathway (35). Therefore, the color of oyster meat was obviously changed by sterilizing in oil at 121°C for 30 min (Supplementary Table 1).

**Table 2:** Instruments color of raw and processed oyster meat according to the CIE Lab color scale.

**Treatments**	**Time (min)**	L^*^	a^*^	b^*^	**ΔE**
Raw	—	66.36 ± 0.52^e^	0.12 ± 0.01^h^	7.86 ± 1.20^g^	—
Steamed	5	73.11 ± 0.11^ab^	2.17 ± 0.13^e^	13.35 ± 0.32^cd^	9.04 ± 0.27^bc^
	10	74.23 ± 1.51^a^	2.54 ± 0.36^e^	13.62 ± 1.12^cd^	10.13 ± 0.63^b^
	15	70.31 ± 0.12^d^	4.45 ± 0.53^bc^	16.89 ± 0.88^b^	10.76 ± 1.00^b^
Boiled	5	73.98 ± 0.11^a^	3.83 ± 0.24^cd^	14.59 ± 0.21^bc^	10.83 ± 0.29^b^
	10	71.73 ± 0.69^c^	3.29 ± 0.28^d^	15.66 ± 0.41^bc^	10.01 ± 0.04^b^
	15	70.35 ± 0.66^d^	3.42 ± 0.53^d^	13.06 ± 0.56^cde^	7.34 ± 0.99^cd^
Sous vide^1^	5	74.31 ± 0.42^a^	1.34 ± 0.76^fg^	10.60 ± 0.30^fg^	8.52 ± 0.19^bcd^
	10	71.85 ± 0.71^bc^	0.92 ± 0.06^g^	10.47 ± 0.56^fg^	6.16 ± 0.39^d^
	15	71.46 ± 0.10^cd^	1.93 ± 0.03^ef^	11.63 ± 0.46^def^	6.60 ± 0.19^cd^
Sterilized (in water)	30	65.35 ± 0.57^e^	4.80 ± 0.01^b^	16.78 ± 3.43^b^	10.19 ± 3.05^b^
Sterilized (in oil)	30	50.35 ± 0.25^f^	11.24 ± 0.33^a^	26.45 ± 1.97^a^	26.96 ± 1.08^a^

L^*^ = lightness; a^*^ = redness; b^*^ = yellowness. Different uppercase letters in the same column indicate significant differences (p < 0.05).

1 *Time indicates the internal temperature of the oyster reached 70°C and stayed at this temperature for 5, 10, and 15 min*.

### Thiobarbituric Acid-Reactive Substances (TBARS)

**Figure 1:**
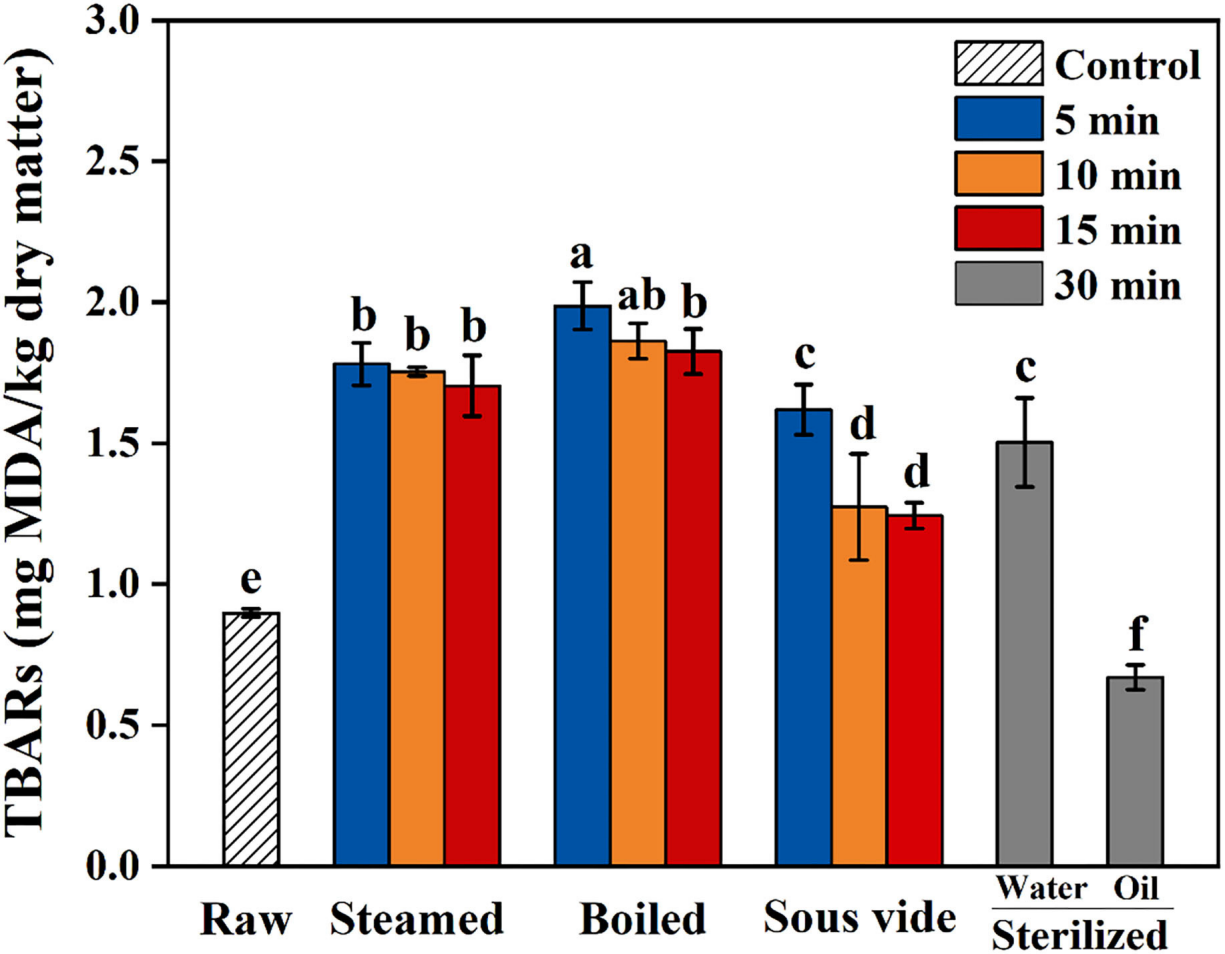
Thiobarbituric acid reactive substances (TBARS) of raw and processed oysters under different thermal processing conditions. Error bars indicate standard deviation (*n* = 3). Samples designated with different lowercase letters were significantly different (*p* < 0.05).

Thiobarbituric acid-reactive substances value is commonly used as an indicator to assess the extent of lipid oxidation in food by measuring the content of MAD (36). TBARS values of oysters under different thermal processing conditions are shown in Figure 1. In the present study, the values of all processed samples were below 2.5 mg MDA/kg dry sample. The values of steamed, boiled, and SV-treated samples were higher than the raw ones and showed similar trends, which gradually decreased with processing time. Compared with raw samples, the value of SW30 increased by 67%, while the value of SO30 decreased by 26%. To the best of our knowledge, the reason for the decrease in SO30 is still unclear. The finding of Tavares et al. (15) was similar to the present study, which reported that the TBARS value of fried filets was about one-half of the boiled ones. We hypothesized that the decrease of TBARS value of oysters processed with a longer time and higher temperature might be caused by the reaction of some aldehydes produced by lipid oxidation with free amino groups of protein *via* Maillard reaction pathways (37).

### GO and MGO Levels

**Figure 2:**
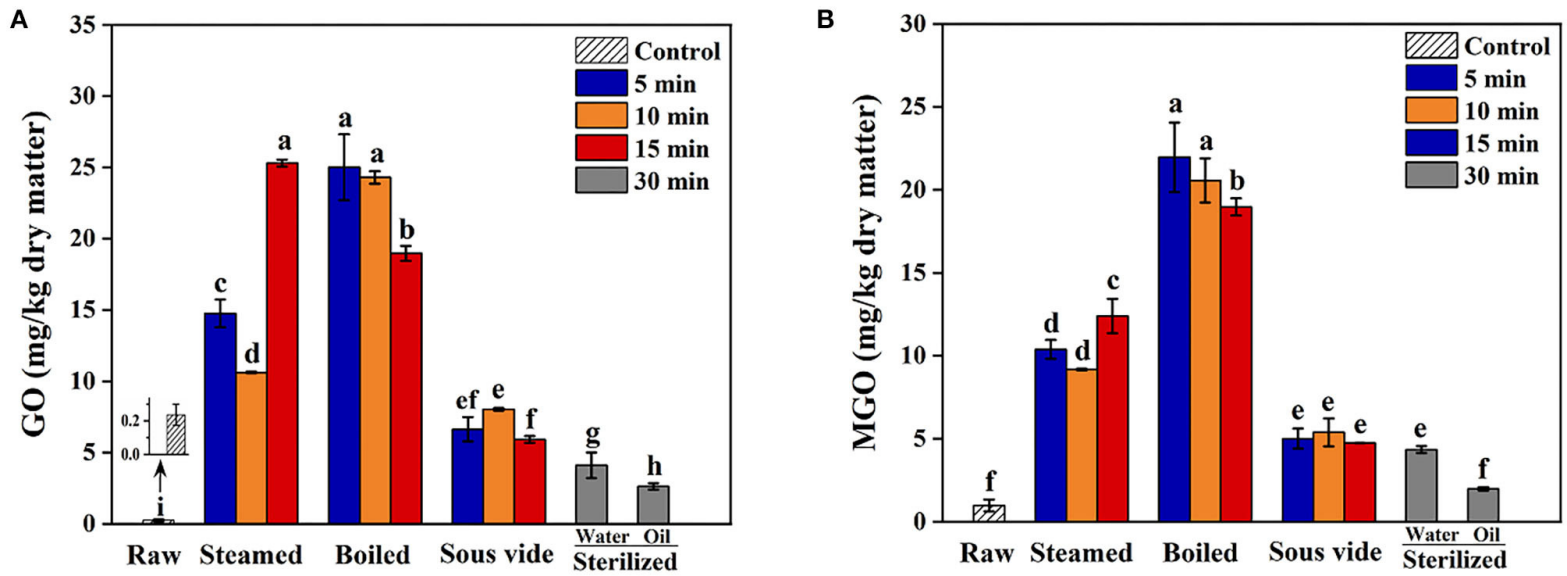
Effect of steamed, boiled, sous vide, and sterilized treatments on the levels of **(A)** glyoxal (GO) and **(B)** methylglyoxal (MGO) in raw and processed oysters. Error bars indicate standard deviation (*n* = 3). Samples designated with different lowercase letters were significantly different (*p* < 0.05).

Glyoxal and MGO levels in oysters under different thermal processing conditions are shown in Figures 2A,B, respectively. All thermal processing methods increased GO and MGO levels of oysters compared to the raw ones, in which the contents of GO and MGO ranged from 2.62 to 25.31 mg/kg dry sample and 1.98 to 21.97 mg/kg dry sample, respectively. The GO and MGO levels of steamed samples increased with processing time, while the boiled ones observed the opposite trend. The SV-treated samples showed relatively lower GO and MGO than the steamed and boiled ones. The phenomenon was probably that mild reaction conditions were not favorable for the production and accumulation of GO and MGO (38). Furthermore, the GO and MGO levels of SO30 were significantly lower than the other processed samples (*p* < 0.05). The research results of Zhu et al. (33) also found that the content of MGO in braised chicken decreased with the increase of sterilization time. Since GO and MGO were the vital substrates in Maillard reactions, we speculated that the substantial reduction of GO and MGO levels in sterilized oysters might be due to the reaction with amino acids to form many Maillard reaction products (38, 39). The mechanisms behind this phenomenon still need to be further investigated.

### Lysine Levels

**Figure 3:**
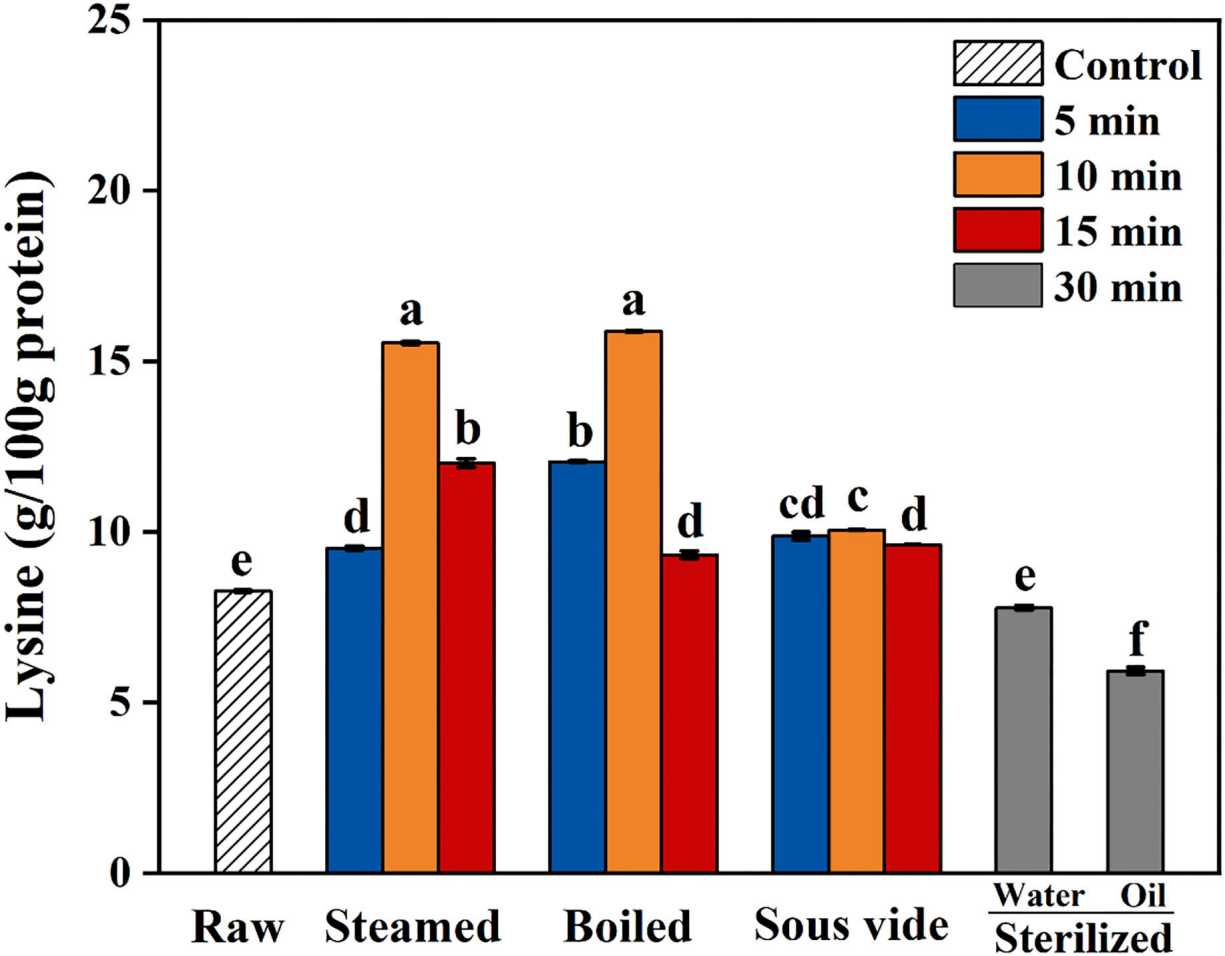
Content of lysine in raw and processed oysters under different thermal processing conditions. Error bars indicate standard deviation (*n* = 3). Samples designated with different lowercase letters were significantly different (*p* < 0.05).

Maillard reaction in food during thermal processing leads to the loss of essential amino acids (especially lysine) and decreased bioavailability (40). The levels of lysine in oysters under different thermal processing conditions are shown in Figure 3. During the first 10 min of steaming and boiling, the lysine levels gradually increased with cooking time. When steaming and boiling for 15 min, the decline of lysine levels were 23 and 41% more than that of 10 min, respectively. Furthermore, the oysters sterilized in water or oil for 30 min had 6 and 28% lower lysine levels than the raw ones, respectively. The decrease of lysine levels could be attributed to the combination of lysine and di-carbonyl compounds, one of the main reactions for CML and CEL formation (33). Regardless of processing time, the levels of lysine in SV-treated oysters were close to that in raw ones. From the present results, the SV treatment could effectively reduce the loss of lysine in oysters than the other processed methods.

### CML and CEL Levels

**Figure 4:**
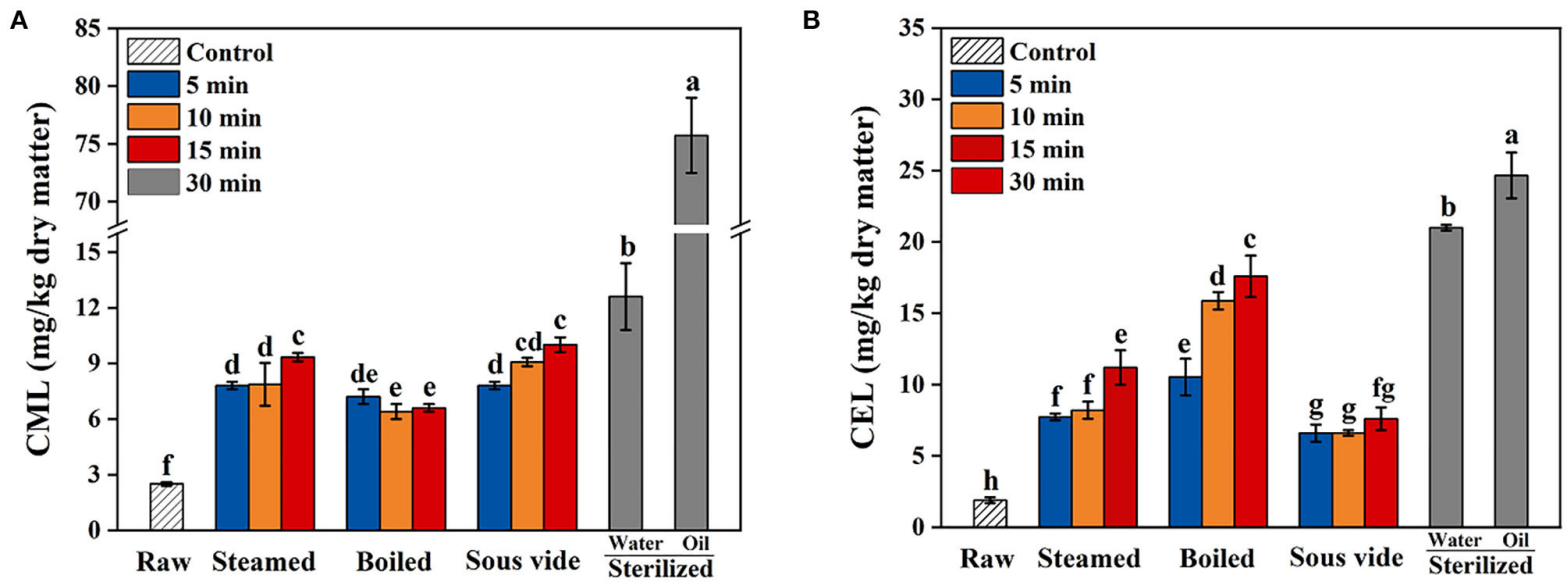
Effects of steamed, boiled, sous vide, and sterilized treatments on the levels of **(A)** *N*^ε^-carboxymethyl-lysine (CML) and **(B)** *N*^ε^-carboxyethyl-lysine (CEL) in raw and processed oysters. Error bars indicate standard deviation (*n* = 3). Samples designated with different lowercase letters were significantly different (*p* < 0.05).

The levels of CML and CEL in oysters under different thermal processing conditions are shown in Figure 4. In general, the levels of CML and CEL in all processed oysters were increased with processing time. As shown in Figure 4A, the levels of CML in steamed, boiled, and SV-treated oysters for 15 min increased by 3.7, 2.6, and 3.6 times that in the raw ones, respectively. Sterilizing in water or oil for 30 min compared to 0 min, the levels of CML increased by 5.0 and 30.3 times, respectively. Therefore, it was summarized that the formation of CML during sterilizing was larger than that in the other three processes. As shown in Figure 4B, the levels of CEL in steamed, boiled, and SV-treated oysters for 15 min increased by 5.9, 9.3, and 3.5 times that in the raw ones, respectively. Sterilizing in water or oil for 30 min compared to 0 min, the levels of CEL increased by 11.1 and 13 times, respectively. The CEL level in oysters processed by SV is the lowest among all methods. Based on the above results, SV treatment could better control CEL increase during thermal processing. To the best of our knowledge, few studies were available on the effect of SV treatment on the formation of AGEs in oysters. So the mechanism behind this phenomenon was not precise, which should be further investigated.

In addition, the CML level in processed oysters was lower than that of CEL except sterilized (in oil) and SV-treated ones. Similar results were found in Sun et al. (13) and Yu et al. (14), which reported that the CEL level in commercial sterilized beef and sterilized meat products was higher than that of CML. However, Zhu et al. (33) found that the content of CML (1.97–27.57 mg/kg) in braised chicken was higher than that of CEL (0.08–0.72 mg/kg). This difference may be caused by different types of food and processing methods, as well as different reaction rates of lysine with GO or MGO (41).

### Correlation Analysis

**Figure 5:**
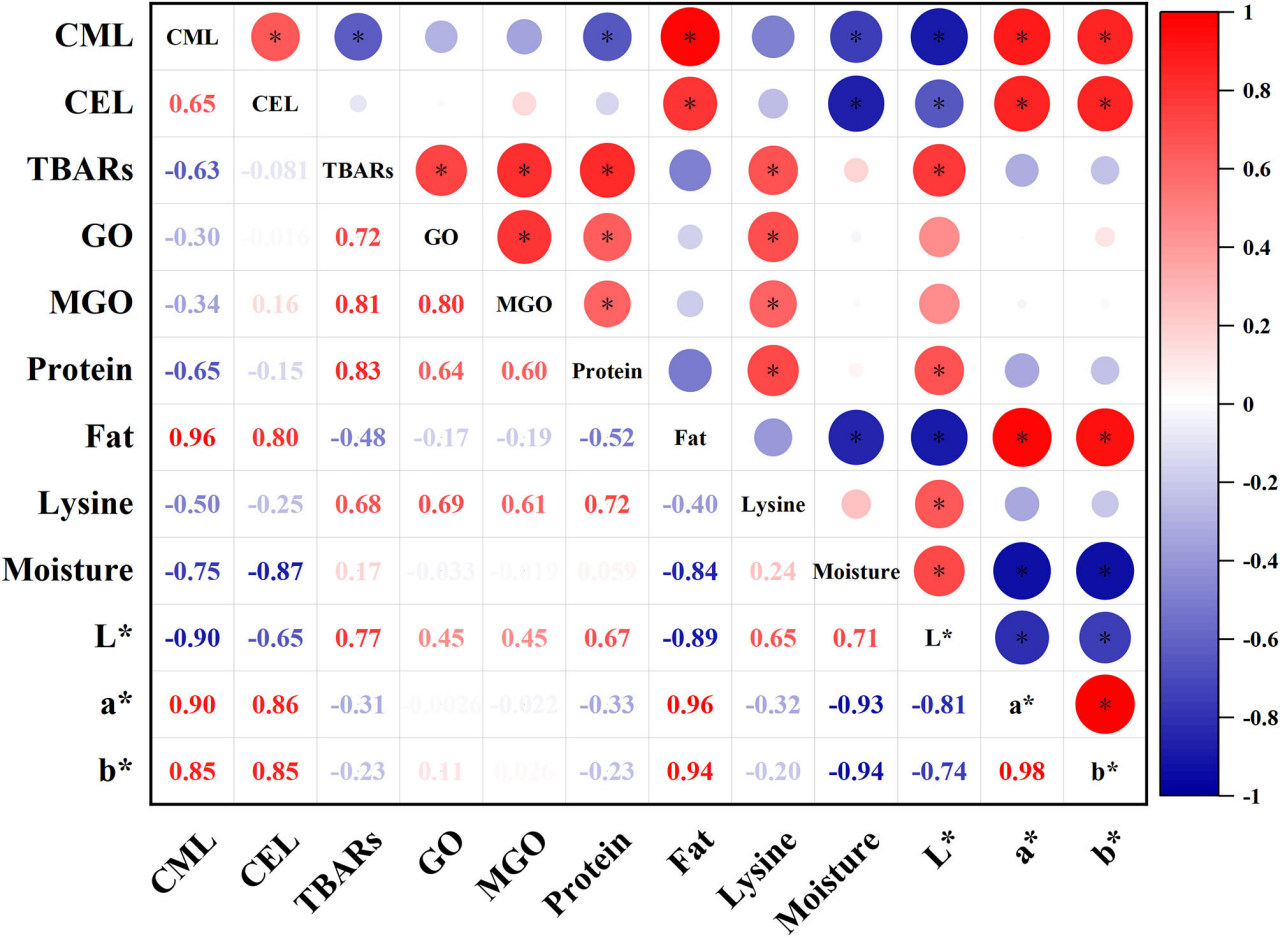
Correlation analysis of all parameters measured in this research of oysters under different thermal processing conditions. Asterisk (*) indicated significant correlation (*p* < 0.05).

To further understand the relationship between composition, oxidation, precursor, and AGEs of oysters during thermal processing, the correlation between them was analyzed (Figure 5). It was observed that the generation of CML and CEL were positively correlated with fat content, a^*^ and b^*^ value (*p* < 0.05), and negatively correlated with moisture content and L^*^ value (*p* < 0.05). Besides, CML was negatively correlated with TBARS and protein content (*p* < 0.05). Interestingly, CML and CEL levels were weakly associated with GO, MGO, and lysine content (*p* > 0.05).

The formation of AGEs is strictly linked with the later steps of the Maillard reaction and is affected by food composition (8, 15). In the present study, high CML and CEL levels in oysters were closely related to the high fat and low moisture content. Similar results were found in evaporated milk, canned salmon, and peanut sauce (11), suggesting that the formation of CML and CEL can be promoted under low-moisture and high-fat conditions (9). Besides, high CML and CEL levels were also closely related to the high a^*^, b^*^, and low L^*^ values. The more severely the oyster meats were processed, the darker it would be. In fact, several studies have shown that many known Maillard reaction products, such as CML and CEL, have distinctive yellow to brown colors (42, 43).

In addition, the influence of lipid oxidation on the formation of AGEs is very complex, and some pathways are still unclear. Our results showed that the TBARS was negatively correlated with the CML level (*r* = −0.63, *p* < 0.05), which indicated that the formation of CML was closely related to the consumption of lipid oxidation productions. Besides, the TBARS was positively correlated with GO level (*r* = 0.72, *p* < 0.05), suggesting that the lipid oxidation caused the formation of some aldehyde compounds, such as GO. However, CML level had weak correlation with GO and lysine content (*r* = −0.3, *p* > 0.05 and *r* = −0.5, *p* > 0.05, respectively). The reason behind this interesting finding might be that some aldehyde compounds generated from lipid oxidation were the vital substrates in Maillard reactions and spontaneously reacted with amino acids under high-temperature conditions (38, 44). Furthermore, CEL level was insignificantly correlated with TBARS, MGO, and lysine content. Based on the results above, it was summarized that the combined effect of lipid oxidation and Maillard reactions during thermal processing of oysters had a greater effect on the formation of CML but less on the formation of CEL.

### *Z*-Score Analysis

**Figure 6:**
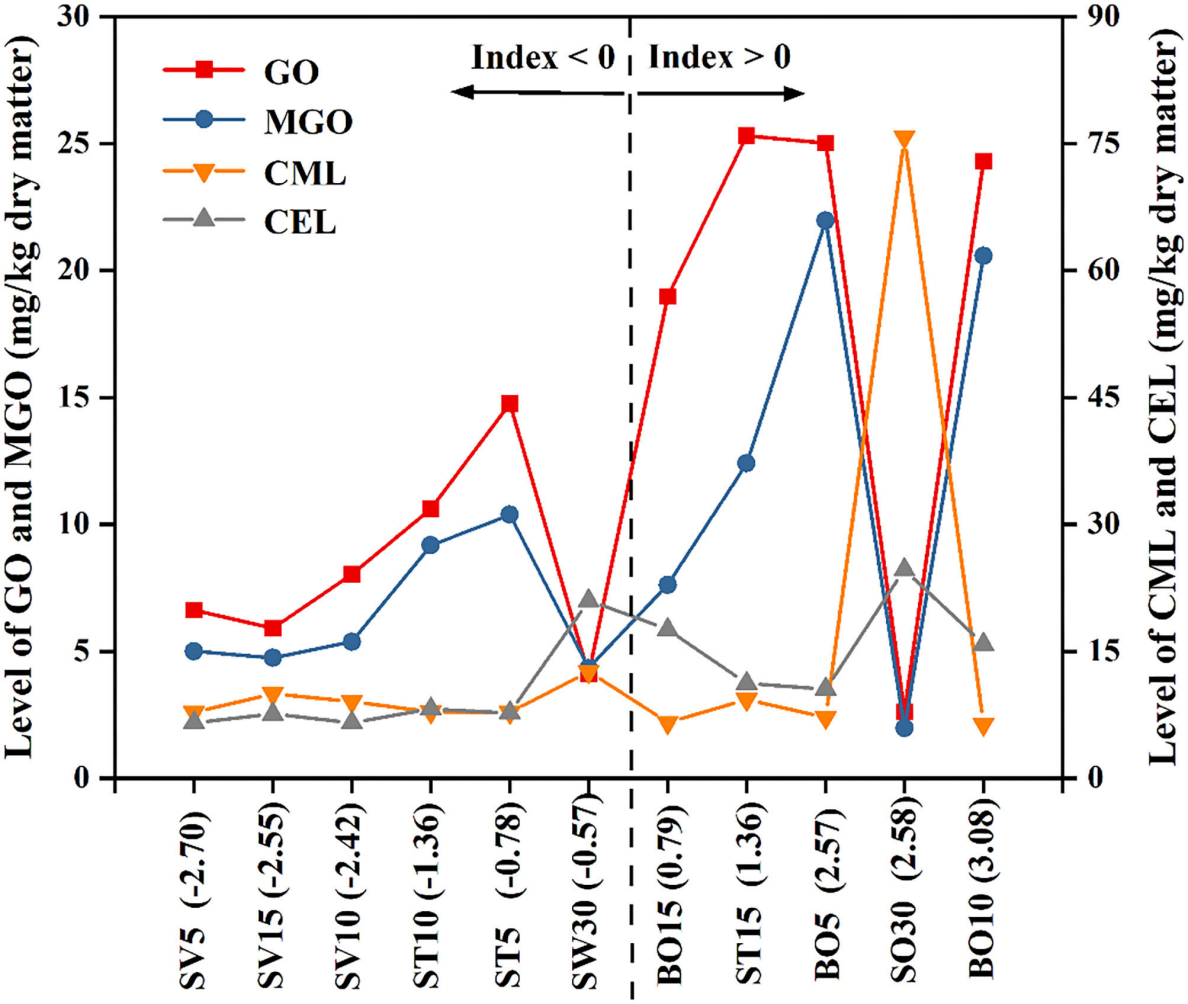
The distribution of various processed oysters based on *Z*-score analysis.

To make a comprehensive comparison of different processed oysters based on their levels of aforementioned di-carbonyl compounds (Figure 2) and AGEs (Figure 4). The *Z*-score and Index value (total *Z*-score) of each processed oyster were calculated, and a graph was drawn to show the level of GO, MGO, CML, and CEL based on the order of Index value (Figure 6). *Z*-score indicates the value of how many standard deviations from the mean, while Index value is the sum of *Z*-scores representing the full derivations in terms of the GO, MGO, CML, and CEL. Therefore, Index < 0 means less than average value, and the less of Index, the better, and vice versa (45). In the present study, the SV-treated oysters (SV5, SV10, and SV15) had lower Index values (−2.7, −2.42, and −2.55, respectively), probably owing to the relatively mild thermal processing conditions which restricted the formation of AGEs and di-carbonyl compounds. However, the Index values of boiled oysters (BO5, BO10, and BO15) were 2.57, 3.08, and 0.79, respectively. During high-temperature sterilization, the Index value of SW30 was −0.57, whereas the Index value of SO30 was 2.58. Based on the above results, endogenous hazardous substances were formed in boiled oysters (BO5, BO10, and BO15) and SO30, with GO or MGO as the major endogenous hazardous substances in boiled oysters and CML or CEL being the predominant one in SO30. In summary, the SV processing (SV5, SV15, and SV10) with the lowest Index value of the four methods mentioned in this study were recommended to consumers.

## Conclusion

In summary, the effect of four thermal processing methods [steaming, boiling, sous vide (SV), and sterilizing] on CML and CEL formation of oysters was first investigated in the present study. The formation of CML and CEL was closely related to high-fat content, a^*^, and b^*^ values as well as low moisture content and L^*^ value. Besides, lipid oxidation during thermal processing promoted the formation of CML but had less effect on the formation of CEL. According to the Z-score of processed oysters, SV treatment at 70°C within 15 min might be an effective thermal processing method to control the formation of CML and CEL. This study could provide some valuable references and guidelines for the safety of home-cooked and commercial sterilized oysters based on the control of AGEs levels.

## Data Availability Statement

The original contributions presented in the study are included in the article/Supplementary Material, further inquiries can be directed to the corresponding author/s.

## Author Contributions

PZ, SD, and MZ involved in conceptualization, involved in writing, and reviewing, and editing. PZ involved in analysis, performed methodology, conducted the investigation, and wrote the original draft. MZ and SD performed funding acquisition and supervised the study. All authors contributed to the article and approved the submitted version.

## Conflict of Interest

The authors declare that the research was conducted in the absence of any commercial or financial relationships that could be construed as a potential conflict of interest.

## Publisher's Note

All claims expressed in this article are solely those of the authors and do not necessarily represent those of their affiliated organizations, or those of the publisher, the editors and the reviewers. Any product that may be evaluated in this article, or claim that may be made by its manufacturer, is not guaranteed or endorsed by the publisher.
