# Supplementary material for: Formation of Nε-Carboxymethyl-Lysine and Nε-Carboxyethyl-Lysine in Pacific Oyster (Crassostrea gigas) Induced by Thermal Processing Methods
Source: Front Nutr. 2022 Apr 15;9:883789. doi: 10.3389/fnut.2022.883789 (PMC9051442; doi:10.3389/fnut.2022.883789)
Supplement: Supplementary file 1 [file Table_1.DOCX]

**Supplementary material**

**Table 1.** Photos of raw and thermal processed oyster meats.

| **Treatments** | **Processing time** | | |
| --- | --- | --- | --- |
| Sterilized  (121°C) | *0 min (Raw)*  *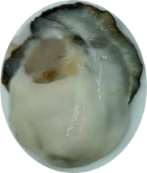* | *30 min (in water)*  *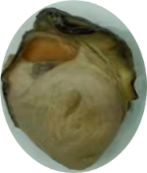* | *30 min (in oil)*  *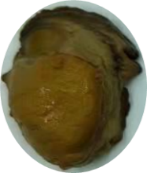* |
| Sous vide ^1^  (70°C) | *5 min*  *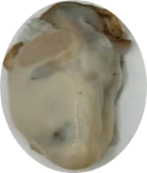* | *10 min*  *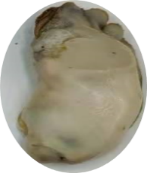* | *15 min*  *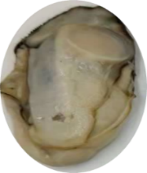* |
| Steamed  (98°C) | *5 min*  *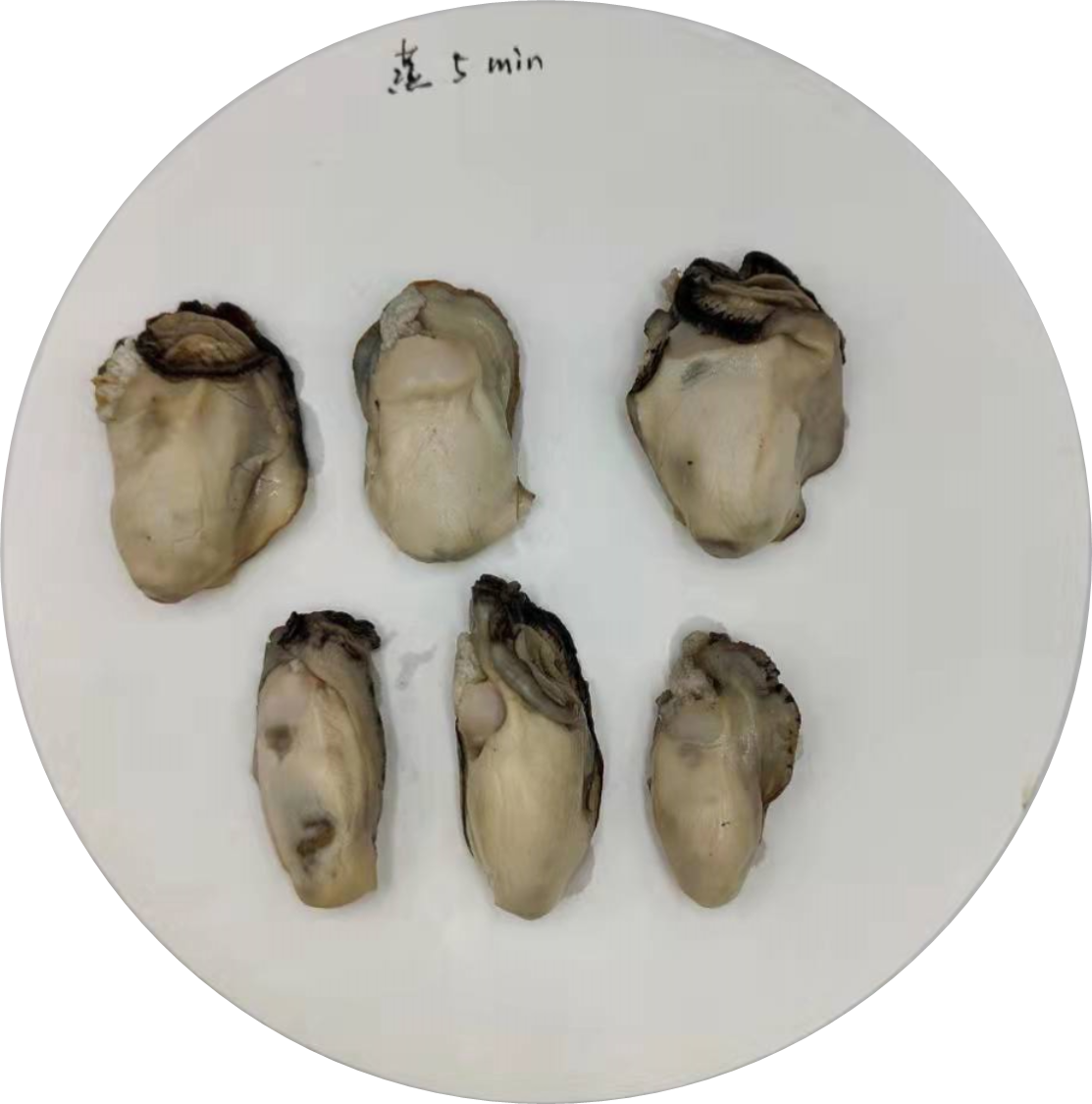* | *10 min*  *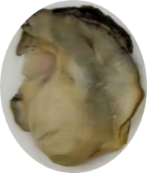* | *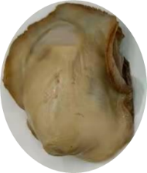15 min* |
| Boiled  (98°C) | *5 min*  *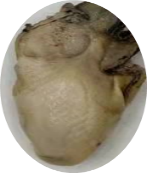* | *10 min*  *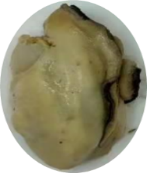* | *15 min*  *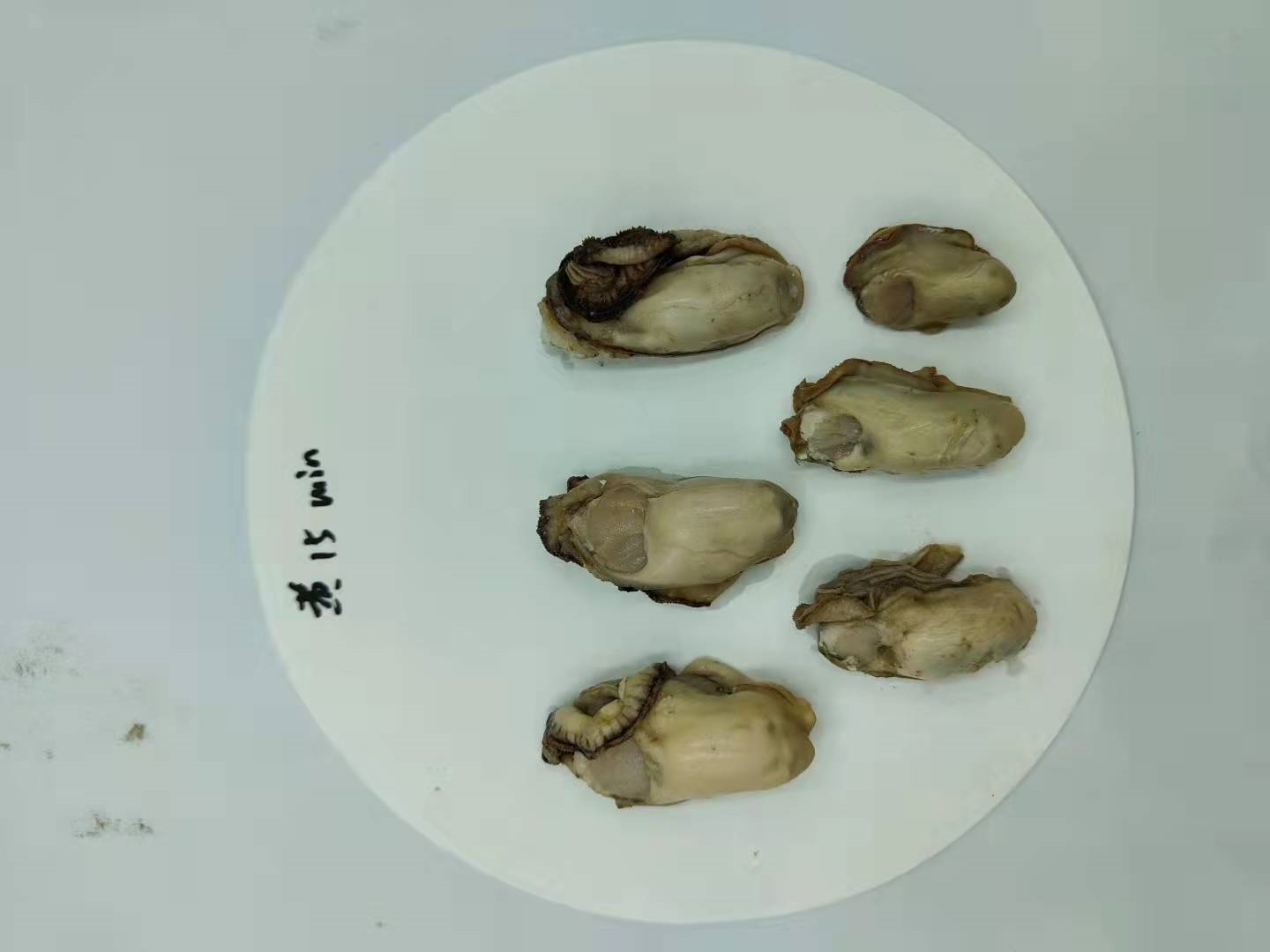* |

Temperatures indicate the thermal processing temperature.

^1^ Time indicate the internal temperature of oyster reached 70°C and stayed at this temperature for 5 min, 10 min and 15 min.
